# Surgical management of scalp cirsoid aneurysms: Kuwait experience. (case series)

**DOI:** 10.1016/j.amsu.2022.103479

**Published:** 2022-03-08

**Authors:** Abdullah A. AlFawaz, Hamad J. AlShatti, Ali H. Safar

**Affiliations:** a Faculty of Medicine, Kuwait University, Kuwait; b Mubarak Hospital, Department of Surgery, Vascular Division, Kuwait; c Mubarak Hospital, Department of Surgery, Kuwait

**Keywords:** Cirsoid aneurysm, Scalp arterio-venous malformation, Surgical excision, Pre-operative embolization

## Abstract

**Background:**

Cirsoid aneurysms are arteriovenous malformations of the scalp region that usually manifest as a painless pulsatile mass. These are present in the younger age group and frequently associated with trauma.

**Objectives:**

Several treatment algorithms have been proposed, and we report our experience with sole surgical management.

**Methods:**

Retrospective review of all the scalp vascular malformation cases performed in the main national Vascular Surgery Service of Kuwait. Pre-operative data, including patient demographics were obtained. All patients underwent diagnostic vascular Duplex ultrasound and angiography. Intra-operative and post-operative data, including outcomes and follow up were recorded.

**Results:**

Six patients with Cirsoid aneurysm, four females and two males, had a mean age of 19.7 years (range, 10–33 years). All the patients presented with a painless pulsating mass in the scalp (4 Anterolateral and 2 posterior), and one case had associated dizziness and headache. These malformations were found to be solely fed by the extra-cranial vessels with no intra-cranial communication. One patient had pre-operative embolization prior to excision, and the rest had sole surgical excision. No postoperatively complications or recurrence were seen at 2–5 year follow up.

**Conclusions:**

Cirsoid aneurysms are amenable to sole surgical excision with excellent results after excluding intra-cranial communication.

## Highlights

• Cirsoid aneurysms are extra cranial AVMs arising in the scalp
• Most patients present with painless mass.
• Diagnosis is made with duplex ultrasound and/or CT angiography.
• We recommend sole surgical over endovascular therapy or a hybrid approach.

## 1 Introduction

Cirsoid aneurysms are rare subcutaneous arteriovenous malformations arising in the scalp region [1]. In the literature, they are also referred to as plexiform angioma, aneurysma sepertinum, and aneurysm racemosum [1,2].

Etiologically, cirsoid aneurysms can be congenital, traumatic, or iatrogenic with the majority of the reported cases being of spontaneous origin [1,2].

Cirsoid aneurysms feeding and drainage systems are usually by extracranial vessels however some reports showed intracranial involvement [3,4]. The superficial temporal, occipital, and posterior auricular arteries were the most common feeding arteries observed [[1], [2], [3]].

The most common symptoms were progressive swellings, headache, local pain, audible bruits, tinnitus, and cosmetic concerns [[1], [2], [3],[5], [6], [7]].

Angiography is considered the gold standard diagnostic modality for cirsoid aneurysms as it helps in the exact demarcation of supplying and draining vessels and also helps in out ruling intracranial involvement [1].

Their location, complex vascularity, high flow shunting, and cosmetic outcomes make the treatment of cirsoid aneurysms challenging. They can be treated with surgical excision, endovascular embolization, or a combination of both.

We are reporting our experience in surgical management of six cases with Cirsoid aneurysms.

## 2 Methods

Retrospective review of all the scalp vascular malformation cases performed in the main national Vascular Surgery Service of Kuwait. Pre-operative data, including patient demographics, clinical presentation, and investigations were all obtained. Existing patient information collected was de-identified and the original data with identifiers was discarded. The study qualified for exempt status and individual patient consent was not required.

All patients underwent diagnostic vascular Duplex ultrasound and angiography. Interrogation of the intra-cranial, as well as the extra-cranial, vasculature was undertaken in all patients. Catheter-based angiography was predominantly used in the earlier cases, however, for the last case, CT angiography was sufficient. Frontotemporal cirsoids (supplied by superficial temporal artery) were termed anterior and occipital cirsoids (supplied by the occipital artery) were termed posterior. Surgical technique involved exposure and ligation of the main feeding artery prior to Cirsoid resection with preservation of the overlying skin paddle. Intra-operative and post-operative data, including transfusion requirements, immediate and long-term outcomes, and follow up were all recorded. This case series has been reported in line with the PROCESS Guideline and is registered in the Research Registry website under the unique registration number 7618 [8,9].

## 3 Results

Six patients with Cirsoid aneurysms, four females and two males, with a mean age of 19.7 years (range 10–33 years) were identified in our patient registry between the years 2005 and 2019. All the patients presented with a painless non-tender pulsating mass in the scalp with a bruit and a thrill. Four had anterior cirsoids two had posterior cirsoids. One case had associated occasional dizziness and headaches that was not attributed to any other cause. No other neurological symptoms were reported such as seizures or other neurological deficits. None of the patients reported a history of head trauma or scalp/cranial procedures. There was no family history of Cirsoid aneurysms or arteriovenous malformations in all patients. None of the patients reported any past medical history and all lab investigations were within normal limits (including platelet levels).

**Fig. 1:**
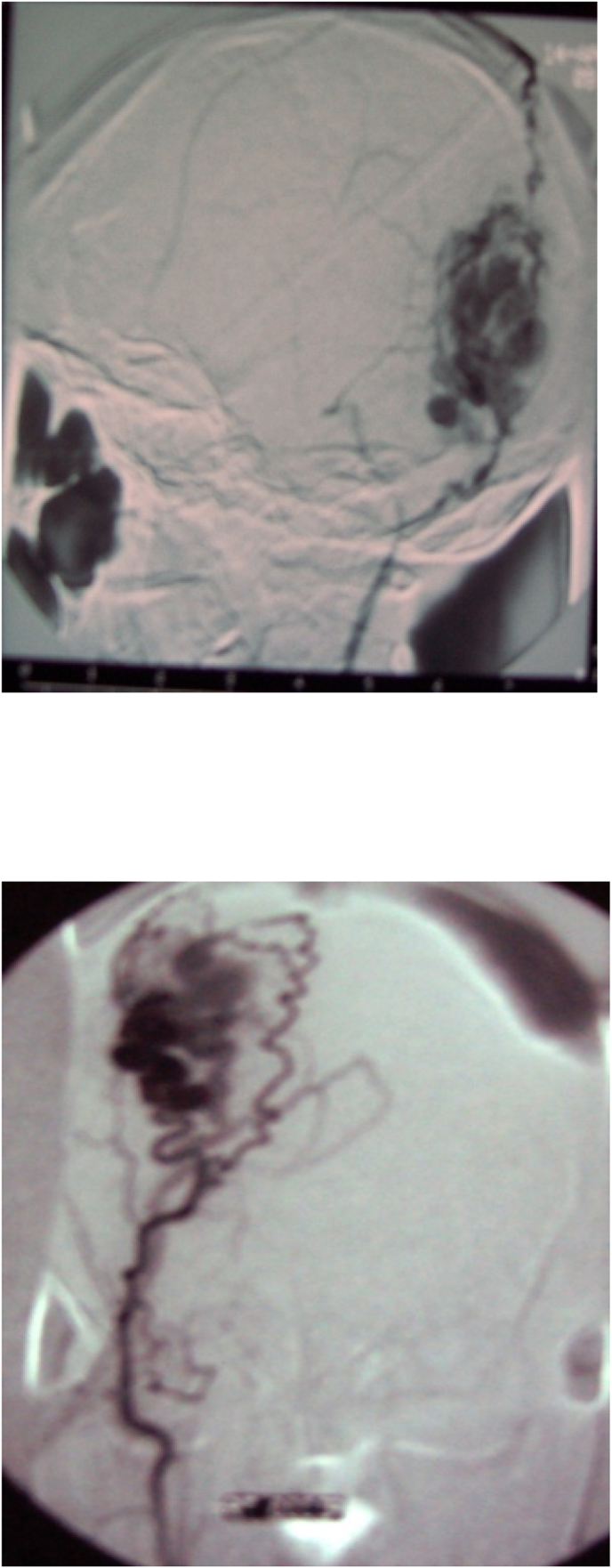
Angiogram shows the extra-cranial feeders of the aneurysm: lateral view A), anterior-posterior view B).

Diagnosis was confirmed with duplex ultrasound and angiography was performed to rule out any intra-cranial vascular communications. Catheter based angiography (Fig. 1) was used for the first five patients and a CT angiogram was used for the last patient. These malformations were found to be solely fed by the extra-cranial vessels with no intra-cranial communication. Four of our patients had the superficial temporal artery as the feeding artery whereas the other two had the occipital artery as the feeding artery. One patient had pre-operative embolization prior to excision, and the rest had sole surgical excision. Pre-operative embolization was performed via femoral access, catheterization of the external carotid artery and selection of the feeding artery (the occipital artery in this case) and injection of Lipiodol/Histoacryl to embolize the artery. This, however, did not completely abolish pulsation within the Cirsoid aneurysm and the patient went on to have the Cirsoid excised in a similar fashion to the other patients.

None of the patients suffered immediate post-operative complications. Blood loss was minimal and no blood transfusions were necessary. A fenestrated drain and compression dressing were used in all patients. On post-operative day 1, drains were removed and patients discharged. There were no wound complications and all incision scars were cosmetically acceptable. No recurrences were seen at 2–5 year follow up. Table 1 summarizes the presentation and management of these patients.

**Table 1:** The demographics and management of the four cases presented.

Cases	1st	2nd	3rd	4th	5th	6th
Demographic						
Age (years)	23	10	22	13	17	33
Sex	Female	Female	Female	Female	Male	Male
Symptoms	mass	mass	mass	mass	mass	mass
Site on Scalp	Posterior	Anterior	Posterior	Anterior	Anterior	Anterior
**Investigations**						
Duplex	+	+	+	+	+	+
Angiogram	+	+	+	+	+	+
**Management**						
Embolization	+	–	–	–	–	–
Surgical Excision	+	+	+	+	+	+
Complication	–	–	–	–	–	–
Recurrence	–	–	–	–	–	–

## 4 Discussion

Cirsoid aneurysms are rare subcutaneous arteriovenous malformations arising in the scalp region [1]. In the Greek literature, Cirsoid is a term that refers to Varyx with Cirsoid aneurysms also known as plexiform angioma, aneurysma sepertinum, and aneurysm racemosum [1,2].

Although named as aneurysms, in reality, Cirsoid aneurysms are fistulas with an abnormal connection between feeding arteries and draining veins [3].

The lesions were found mostly on the Frontal region followed by the Temporal and Occipital regions [1]. Our study contained six patients presenting with Cirsoid aneurysm where four had it on the frontotemporal region and two on the occipital region.

Cirsoid aneurysms can be congenital, traumatic, or iatrogenic with the majority of the reported cases being of spontaneous origin [1,2].

The pathogenesis is yet to be unclear however multiple hypotheses were established. There are 3 hypotheses to explain the pathogenesis of the congenital Cirsoid aneurysms with the first one suggests that Cirsoid aneurysms arise from persistent embryological arteriovenous communication with associated capillary agenesis. The second hypothesis suggests that congenital Cirsoid aneurysms arise from vascular hamartomas and the third hypothesis suggests that at the arteriovenous crossing sites, a fistula is formed. The laceration and the disruption theories were introduced to explain the mechanism behind Traumatic cirsioud aneurysms. The laceration theory suggests that a fistula forms because of a simultaneous laceration between an artery and a vein. However, the disruption theory suggests that the vasa vasorum during trauma ruptures and activates a cellular reaction within endothelial cells and thus creating vascular connections between arteries and veins. The iatrogenic cases were thought to arise at sites of previous interventions such as hair transplantations and craniotomies [1,[4], [5], [6]]. Among the six patients included in our study, none have reported a history of trauma and most likely their presentation is explained by one of the congenital hypotheses.

The most common symptoms were progressive swellings, headache, local pain, audible bruits, tinnitus, and cosmetic concerns [[1], [2], [3],[5], [6], [7]]. All patients included in our study reported swelling and only one patient out of six had associated headache and dizziness. The growing nature of Cirsoid aneurysms manifests by the presence of high flow arteriovenous fistula resulting in blood shunting and progressive dilation and enlargement of draining veins [1].

The vascular origins are usually unconnected to the intracranial or cerebral vasculature. In rare cases, the aneurysm feeding and drainage were by intracranial vessels such as the pial arteries and transosseous emissary veins [10]. The superficial temporal artery was the major feeding vessel in the majority of the reported cases in the literature and this is thought to be due to its superficial location making it vulnerable to trauma. In addition to that, other case reports showed other vessels acting as feeders such as the occipital and the posterior auricular arteries [1,2,4,5,11].

Four of our patients had the superficial temporal artery as the feeding artery whereas the other two had the occipital artery as the feeding artery.

The differential diagnoses for Cirsoid aneurysms include temporal artery aneurysms, Hamartomas, and Hemangiomas [2,5].

Ultrasonography is an initial diagnostic modality that is used initially to out rule vascular injuries resembling swellings, especially in a traumatic setting [12]. Angiography is considered the gold standard modality for diagnosing Cirsoid aneurysms. It allows accurate demarcation and identification of the feeding arteries and draining veins and with the complex vascular anatomy of the fistula and thus out ruling other differential diagnoses. While angiograms are invasive, Computed tomography of the head and Magnetic resonance studies are considered a very effective alternative that produces a comparable (to angiograms) structuring of the aneurysm with low associated risks [1].

All patients in our study had Ultrasonographic scanning. Catheter based angiography was used for the first five patients and a CT angiogram was used for the last patient.

Cirsoid aneurysms are considered to be challenging due to the nature of their location, complex vascularity, high flow shunting, and cosmetic outcomes. They can be treated with surgical excision, endovascular embolization, or a combination of both.

Surgical excision is the classic approach for Cirsoid aneurysms treatment.

Its lower rate of complications and recurrence, effective and immediate resolution of symptoms, cost-effectivity, and satisfying cosmetic outcomes allows the surgical approach to be a convenient and appropriate [3,5,7,13,14]. Perioperative bleeding, necrosis, infection, and recurrence following incomplete resection are the most common complications of surgical intervention [2,3,6,15]. Initial ligation of all feeding arteries and complete resection showed to minimize intraoperative bleeding and produce satisfactory outcomes [5]. Elkiran et al. reported the use of a half-moon flap to include the entire lesion and to avoid the use of unhealthy skin. The half-moon flap helped in disrupting the flow of collaterals and this prevented recurrence. In case of the presence of necrotic skin, a rotational advancement flap was used to replace the necrotic skin and this produced acceptable results with reduced morbidity [7]. En bloc excision is a surgical method used by El shazly et al. and Sousa et al. for highly vascular lesions with thin skin and both reported satisfying results [2,14]. Four patients were treated with surgical excision alone after ligation of all feeding vessels. Intra-operative blood loss was minimal and none received blood transfusion.

Endovascular embolization techniques are minimally invasive can be used alone or in combination with surgical excision. The endovascular embolization can be done through transarterial and transvenous routes, direct puncture, or both. The use of embolic materials such as coils, Onyx, isobutyl-2-cianocrylate, n-butyl cyanoacrylate, polyvinyl alcohol particles, Eudragit-E, and thrombin helps in the obliteration of the vessels [2].

The main advantage of the endovascular approach is that it is less invasive and quicker however it has its pitfalls. Endovascular embolization carries a higher risk of recurrence as not all blood vessels can be processed during the procedure. It also has a high rate of complications such as scalp pain, hair loss, necrosis over the lesion, hyperemia over the skin, and embolization into general circulation. Surgery was also needed following endovascular interventions mainly for tenderness after embolization [14].

The complication rate observed in patients treated by endovascular embolization (55.8%) was significantly higher than those treated surgically (9.9%) according to Sofela et al. [1] Scalp necrosis (5.7%), wound infection (2.1%), hemorrhage (1.4%) were the most common complications associated with surgical excision, whereas transient scalp pain (34.6%), residual mass (13.5%) and painless skin inflammation were mostly associated with endovascular embolization [1].

Another treatment option is endovascular embolization combined with surgical excision. Embolization as a neoadjuvant therapy before surgical excision reduced vascularity and allows accurate demarcation of the feeding artery with very satisfying outcomes [[5], [15], [16]]. [[,^15^] [[,^16^].

Although it carries a low recurrence and complications rate, Sofela et al. states that there was no significant difference in complication and recurrence rates when comparing combination therapy to surgical excision if done in a well planned manner [1,14].

The risks associated with endovascular access and angiography before embolization includes contrast-induced nephropathy, access site complications, and cerebral embolization [17].

The first case in our study was treated first with intra-arterial embolization with Histoakryl/Lipiodol to thrombose the main feeding arteries to the aneurysm, but it did not completely abolish the pulsation, the patient subsequently underwent surgery like the other four patients. As mentioned previously, not being able to process all the feeding arties can be the reason behind not abolishing the pulsation, and hence requiring surgical intervention.

Based on the above risks, the similarity in complications and recurrence rates, and the satisfying outcomes of our patient cohort we concluded that surgical excision is the treatment of choice for cirsoid aneurysms as it offers a complete resolution of symptoms, good cosmetic outcomes, and low complication and recurrence profiles.

## 5 Conclusion

Scalp cirsoid aneurysms are rare. Angiography (catheter based or CT) is recommended to exclude intra-cranial extension of the aneurysm, and sole surgical excision is the treatment of choice.

## Ethical approval

None. Pre-operative data, including patient demographics, clinical presentation, and investigations were all obtained. Existing patient information collected was de-identified and the original data with identifiers was discarded.  The study qualified for exempt status and individual patient consent was not required.

## Sources of funding

None.

## Author contribution

Contributor 1: Conception/design, Data Acquisition, Data Analysis/Interpretation, Manuscript Writing, Critical Review.

Contributor 2: Data Acquisition, Data Analysis/Interpretation, Critical Review.

Contributor 3: Data Analysis/Interpretation, Manuscript Writing, Critical Review.

## Consent

Existing patient information collected was de-identified and the original data with identifiers was discarded. The study qualified for exempt status and individual patient consent was not required.

## Registration of Research Studies

Name of the registry: Research Registry
Unique Identifying number or registration ID: 7618
Hyperlink to your specific registration (must be publicly accessible and will be checked): https://www.researchregistry.com/browse-the-registry#home/registrationdetails/620002b51d3c97001ef43008/

## Guarantor

Dr. Abdullah A. AlFawaz.

Address: Mubarak Al-Kabeer Hospital - Kuwait.

Phone numbers: 965-24636213.

Facsimile numbers:

E-mail address: abdullah.alfawaz@HSC.EDU.KW.

## Provenance and peer review

Not commissioned, externally peer reviewed.

## Declaration of competing interest

None.
